# Synthetic Calcium Silicate Biocomposite Based on Sea Urchin Skeleton for 5-Fluorouracil Cancer Delivery

**DOI:** 10.3390/ma16093495

**Published:** 2023-05-01

**Authors:** Evgeniy K. Papynov, Oleg O. Shichalin, Olesya V. Kapustina, Igor Yu. Buravlev, Vladimir I. Apanasevich, Vitaly Yu. Mayorov, Alexander N. Fedorets, Alexey O. Lembikov, Danila N. Gritsuk, Anna V. Ovodova, Sofia S. Gribanova, Zlata E. Kornakova, Nikolay P. Shapkin

**Affiliations:** 1 Department of Nuclear Technology, Far Eastern Federal University, 10 Ajax Bay, Russky Island, 690922 Vladivostok, Russia; papynov@mail.ru (E.K.P.);; 2 Department of Oncology and Radiation Therapy, Pacific State Medical University, 2, Ostryakov Aven., 690990 Vladivostok, Russia

**Keywords:** calcium silicate, wollastonite, sea urchin skeleton, microwave synthesis, hydrothermal synthesis, drug delivery system

## Abstract

Synthetic calcium silicates and phosphates are promising compounds for targeted drug delivery for the effective treatment of cancerous tumors, and for minimizing toxic effects on the patient’s entire body. This work presents an original synthesis of a composite based on crystalline wollastonite CaSiO_3_ and combeite Na_4_Ca_4_(Si_6_O_18_), using a sea urchin *Mesocentrotus nudus* skeleton by microwave heating under hydrothermal conditions. The phase and elemental composition and structure of the obtained composite were studied by XRF, REM, BET, and EDS methods, depending on the microwave heating time of 30 or 60 min, respectively, and the influence of thermo-oxidative post-treatment of samples. The role of the sea urchin skeleton in the synthesis was shown. First, it provides a raw material base (source of Ca^2+^) for the formation of the calcium silicate composite. Second, it is a matrix for the formation of its porous inorganic framework. The sorption capacity of the composite, with respect to 5-fluorouracil, was estimated, the value of which was 12.3 mg/L. The resulting composite is a promising carrier for the targeted delivery of chemotherapeutic drugs. The mechanism of drug release from an inorganic natural matrix was also evaluated by fitting its release profile to various mathematical models.

## 1. Introduction

The use of antitumor (anticancer) drugs (chemotherapy, hormonal, and biological therapy) is a broad current choice for the treatment of metastatic cancer. In this case, every affected organ in the human body is reached through the bloodstream [1]. Chemotherapeutic drugs are based on toxic compounds that primarily inhibit the rapid proliferation of cancer cells. Unfortunately, these drugs also have negative (toxic) effects on healthy cells and the whole body, causing such side effects as cardiocytotoxicity, nephrotoxicity, myelosuppression, neurotoxicity, hepatotoxicity, gastrointestinal toxicity, mucositis, and alopecia [2,3,4,5,6]. Antitumor drugs were shown to cause cardiovascular and renal diseases [7,8,9], dyslipidemia [3], upper limb neuropathy [10,11], and affect patients’ cognitive functions [12], white matter microstructure of the brain, bone tissue parameters, etc. Indiscriminate destruction of cells and toxic side effects of chemotherapeutic agents was, for many years, the only possible approach to the treatment of metastatic disease.

This unspecific, and far from ideal, strategy changed with the discovery of various new targeted drug delivery systems. In particular, drug delivery systems in the form of inorganic and organic materials, which are drug carriers, are widely researched and already used in practice. Carriers can be polymeric nanoparticles, dendrimers, carbon nanoparticles, inorganic nanoparticles, micelles, magnetic nanoparticles, bioresorbable inorganic materials, etc. [13]. These materials interact with the drug by the principle of physical and chemical processes (chemical sorption, molecular exchange, physical immobilization, electrostatic interaction, etc.). They firmly hold the drug and deliver it to the affected organ through blood, directed surgery, etc. [14]. After that, the drug is released, and the carrier is either excreted naturally or dissolved in the body, without causing any adverse effects. This therapy minimizes the penetration of chemotherapeutic drugs into healthy tissues and organs that are not affected by cancer, which reduces the high toxic load on the entire body.

Important antitumor drugs are cisplatin [15], paclitaxel [16], and the widely used 5-fluorouracil [17,18], which belongs to the group of pyrimidine structural analogs. It is used as an antitumor agent to treat cancer of the esophagus, stomach, pancreas, biliary tract, head and neck, liver, and uterine cervix. The drug 5-Fluorouracil exists in four tautomeric forms and has two potential deprotonation centers. The tautomer is fundamental to the analysis of protonated and deprotonated forms of 5-fluorouracil, because it demonstrates the greatest stability. The presence of negative functional groups, and the aforementioned properties of 5-fluorouracil and the suitably small size of its molecules, allow this drug to sorb efficiently on the active centers of inorganic carriers and penetrate the drug molecules into the material pores, which was proved in a number of studies [19,20,21,22]. This determines the effective use of 5-fluorouracil, together with inorganic matrices, for targeted drug delivery.

Calcium phosphates [23], or, more often, hydroxyapatite (HAP) [24,25,26,27,28] and its composite forms [29], are examples of inorganic carriers of these drugs, in particular, 5-fluorouracil, which are widely studied by scientists. First, they show the proven absolute biocompatibility of these systems with the living organism [27,29,30,31,32]. Second, they have the ability for a prolonged release of the absorbed drug [33,34]. The mechanism of 5-fluorouracil binding to calcium phosphates is explained by its intercalation into the carrier via anion exchange between the negative ions of the drug and the matrix surface, as well as by its adsorption on the free outer surface of the carrier. Especially at high pH values, when the deprotonated form of 5-fluorouracil predominates, an electrostatic attraction occurs between Ca^2+^ ions and 5-fluorouracil, which enhances their interaction [35]. In addition, the ability to sorb drugs is also determined by the structure of the carrier (porosity, particle size). In this regard, a drug carrier is a compound that is biologically compatible with a living organism, and has a certain set of physical and chemical characteristics and properties.

The present work proposes to consider and study the possibility of using a composite compound based on calcium silicates, which includes wollastonite, as a 5-fluorouracil delivery agent. Such works are limited in the literature. The promising use of synthetic wollastonite for this biomedical application is determined by its high biocompatibility, which was proved in the works [36,37,38,39], including our studies on in vitro and in vivo models [40,41] that evaluated its composite forms [42]. Second, the chemical composition of wollastonite is characterized by the presence of Ca^2+^ ions, which will ensure the initiation of sorption interaction with 5-fluorouracil and its subsequent intercalation into the porous structure of the carrier. Third, the ability to actively resorb wollastonite, which can be regulated by the presence of additional calcium silicate phases in its composite composition, will provide a controlled excretion of the drug in the body, when in contact with the affected cells. In order to obtain this composite material, an original synthesis, based on the use of sea urchin skeleton as a raw component, is proposed in this work. The sea urchin skeleton is used for the formation of calcium silicates, and the formation of their porous structure, in the form of an inorganic carrier framework.

In this connection, the aim of this work was the microwave synthesis in hydrothermal conditions of a composite based on crystalline wollastonite and combeite, using a sea urchin skeleton, including the study of its physical and chemical characteristics and evaluation of sorption properties in relation to 5-fluorouracil.

The synthetic material may represent a great prospect for use as an inorganic carrier of chemotherapeutic drugs obtained in a resource-saving way, on the basis of chemically pure biogenic raw materials.

The innovative hypothesis of the application was that drug-loaded nanoparticles could provide sustained release of therapeutic doses of 5-fluorouracil to prevent tumor recurrence after resection. In addition, the mechanism of drug release from the inorganic sea urchin shell was evaluated by fitting its release profile to various mathematical models [43,44,45]. These models have been widely used in the literature to simulate the release profiles of different types of drugs from different delivery agents [45,46,47]. They are (1) the Higuchi square root time model, which was used to model the mechanism of diffusion-controlled drug release from a porous matrix, (2) a first-order time model, which described drug release from a system where the release rate was concentration-dependent, (3) a Baker-Lonsdale model, which expressed the diffusion-controlled mechanism of drug release from spherical matrices, and (4) a Hixson-Crowell cubic root model, which was used to model the drug release profile of dissolution systems. The research protocol was authorized by an interdisciplinary ethics committee of Pacific State Medical University, No. 3, from 16 November 2020.

## 2. Experimental Part

### 2.1. Materials

Skeleton of sea urchin *Mesocentrotus nudus* (Pacific Ocean, Sea of Japan), sodium metaxylate (Na_2_SiO_3_∙5H_2_O), 5-fluorouracil (C_4_H_3_FN_2_O_2_). All reagents used have high purity, with a key component content of 99.999%.

### 2.2. Synthesis Method

Wollastonite was obtained by microwave hydrothermal synthesis, according to the following scheme: a 33% sodium metasilicate solution was added to a sample of sea urchin skeleton, preheated at 800 °C for 1 h in the air in a muffle furnace, and placed in a hydrothermal reactor. Next, microwave heating of the reactor was performed at 180 °C for 30 and 60 min, respectively, on a flexiWAVE unit (power 950 W, frequency 2450 MHz). The precipitate was then filtered and washed with distilled water, followed by thermo-oxidative calcination at 800 °C, for 1 h in the air.

The reaction equation for the formation of synthetic wollastonite is:

4CaCO_3_ + 6Na_2_SiO_3_ → Na_4_Ca_4_Si_6_O_18_ + 4Na_2_CO_3_ (1)

### 2.3. Determination of the Sorption Capacity of Wollastonite, with Respect to 5-Fluorouracil

A sample of 10 mL of 5-fluorouracil solution, at a concentration of 20 mg/L, was placed in a 15 mL plastic cone-shaped tube. Then, samples of 20, 100, and 500 mg of the obtained composite powder (100–200 μm fraction) were added. The solutions were stirred for 64 h. The content of 5-fluorouracil was determined spectrophotometrically, using a Shimadzu UV mini-1240 (Kyoto, Japan) unit at the maximum light absorption for 5-fluorouracil at 265 nm.

### 2.4. Characterization Methods

Phase identification was conducted by means of XRD on a diffractometer D8 Advance “Bruker AXS” (Bruker, Bremen, Germany), using CuKα-source, Ni-filter, angle range 10–80°, scanning step 0.02°, and scanning rate 5°/min. Specific surface area was measured by low-temperature nitrogen physisorption at 77 K on an automated gas sorption analyzer, Autosorb IQ “Quantachrome” (FL, USA), and results were analyzed at the level of BET and DFT models. Surface imaging of the fabricated samples was done by means of SEM on a CrossBeam 1540 XB “Carl Zeiss” microscope (Germany), with EDX add-on by Bruker (Bremen, Germany).

### 2.5. Analysis of Drug Release Kinetics

The mechanism of drug release from nanoparticles was evaluated by fitting the release data to the following mathematical models [26,27,28]:

(1) the Higuchi model:

$$Q=k_{1}t^{0.5}$$

(2) first-order time model:

$$\ln\left(1-Q\right)=-k_{1}t$$

(3) Baker-Lonsdale model:

32[1−(1−Q)23]−Q=k3t

(4) Hixson-Crowell model:

(1−Q)13=−k4t 

where *Q* is proportion of drug released in time *t*; *k_1_*, *k_2_*, *k_3_*, *k_4_* are release rate constants, which were obtained by fitting the drug release profile to (1), (2), (3), and (4), respectively. A linear regression analysis of the dissolution data was then performed using Microcal (MT) origin version 6, and a straight line was drawn through the data. The slope of this line gives a constant release rate. In addition, the correlation coefficient (Rc) was determined, which is a statistical measure of how closely the regression line approximates the real dissolution data.

### 2.6. Investigation of the Powder Resorption in the Ringer’s Solution

The resorption of CaSiO_3_ powders was determined by a change in the time of Ca^2+^ concentrations in the Ringer’s solution. The powder was soaked in the solution, with a powder/liquid ratio equal to 0.5/50 g/mL, at 37.5 °C, under static conditions. At certain time intervals (from 1 to 7 days), Ca^2+^ concentration in solutions were determined using methods of chemical analysis. To maintain a constant volume, a portion of the fresh Ringer’s solution was added to the system each time, after the liquid part was taken away for analysis. After soaking, the samples were filtered, washed on the filter by two portions of distilled water (50 mL each), and dried at 90 °C. The weight of the undissolved part of the powders was measured, and weight loss was calculated.

### 2.7. Release of the Drugs

An amount of 500 mg of pre-saturated sorbent was placed in 10 mL of a solution of slightly acidic medium (pH = 5), imitating the pH of the tumor affected area. The release of the drugs was checked at selected time intervals: 0.08 h, 0.33 h, 0.66 h, 1 h, 2 h, 3 h, 4 h, 5 h, 6 h, 24 h, and then every 24 h, until the 5-fluorouracil analytical signal was stopped. During these time intervals, the mixtures were stirred gently at 37 °C in a tube rotator. In each defined time interval, the suspension was centrifuged, and the supernatant was removed and analyzed. The content of the drug was determined by the photometric method, according to the graduation chart, using a UV mini-1240 spectrophotometer (Shimadzu, Japan) at the maximum light absorption for 5-fluorouracil—265 (266) nm.

## 3. Discussion

The use of a sea urchin skeleton for the synthesis of composite material based on calcium silicates is determined by the fact that this material represents a raw material (source Ca^2+^) for the formation of the necessary chemical composition; in particular, wollastonite. Second, it represents a matrix (template) for the formation of a porous inorganic skeleton in the composite. A similar solution has already been successfully implemented by the authors of the present study, earlier in [48,49], where biomimetic composites were synthesized. In the present study, a simpler method of reaction, according to Equation (1), implemented under microwave hydrothermal heating conditions, was chosen. This resulted in the dispersed inorganic synthesis product described below.

**Figure 1:**
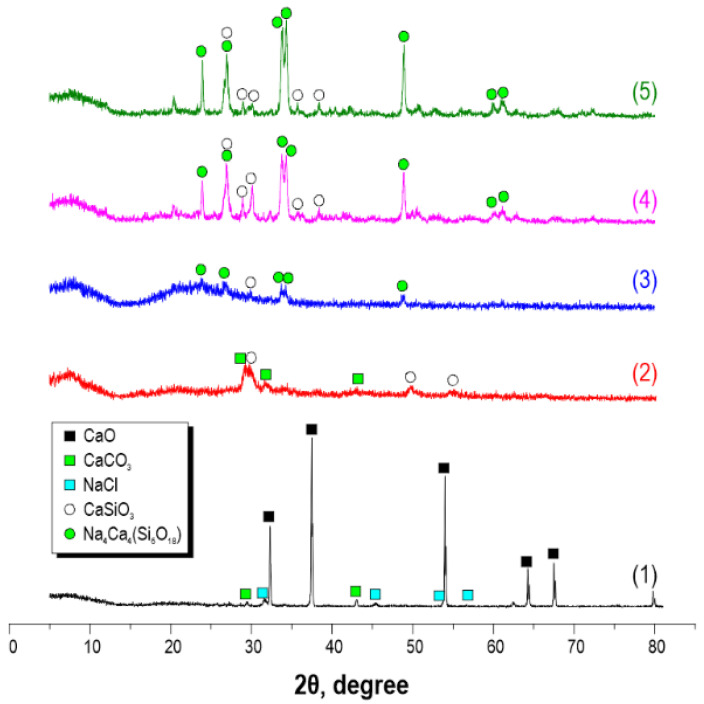
X-ray patterns of material samples obtained from the sea urchin skeleton: (**1**) initial sea urchin skeleton calcined at 800 °C in the air; (**2**) sample after 30 min microwave treatment; (**3**) sample after 60 min microwave treatment; (**4**) sample (**2**) after additional 800 °C thermal oxidation treatment; (**5**) sample (**3**) after additional 800 °C thermal oxidation treatment.

According to XRF data, after thermal-oxidative treatment of the sea urchin skeleton at 800 °C, the material composition includes two main crystal phases of CaO and CaCO_3_, as well as a small amount of NaCl impurity (Figure 1, curve 1). The presence of sodium chloride as an impurity is explained by the sample preparation of the sea urchin. After microwave treatment of the sea urchin skeleton, the sample is saturated with Na_2_SiO_3_ solution at 180 °C and 30 min of exposure, and the sample composition becomes a mixture of CaCO_3_ and crystal wollastonite CaSiO_3_ (Figure 1, curve 2). When the time of exposure in microwave heating conditions increases up to 60 min, the composition of the obtained sample, along with the crystal wollastonite CaSiO_3_ phase of sodium calcium aluminosilicate Na_4_Ca_4_(Si_6_O_18_) of combeite structure, is formed, and CaCO_3_ is absent (Figure 1, curve 3). The subsequent thermal-oxidative treatment of the synthesized materials is accompanied by an increase in the degree of crystallinity of CaSiO_3_ wollastonite and Na_4_Ca_4_(Si_6_O_18_) combeite, as evidenced by the increased intensity of the diffraction maxima (Figure 1, curves 4 and 5).

**Figure 2:**
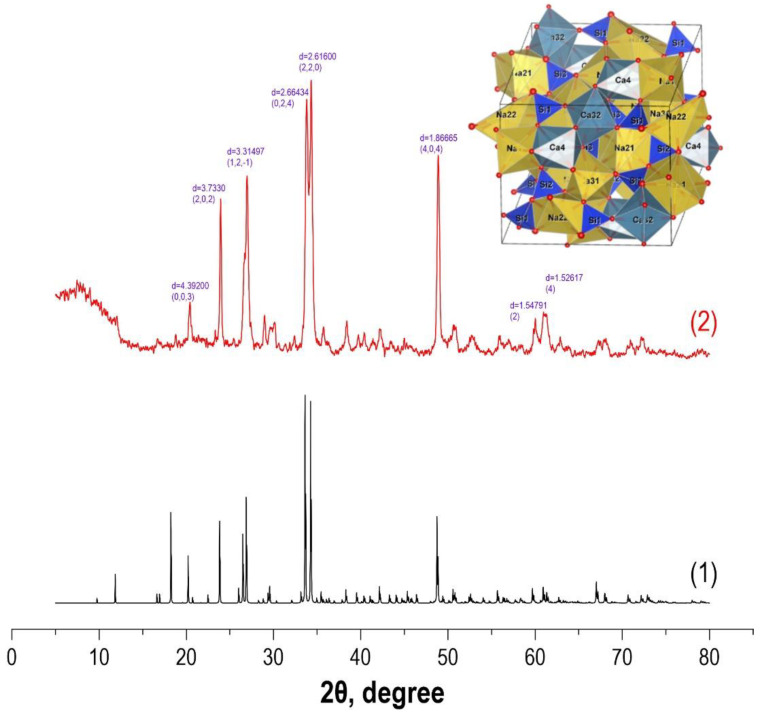
XRD comparisons of 180 °C experimental sample with reference structures: (**1**) Na4Ca4Si6O18 phases; (**2**) sample after 30 min microwave treatment after additional 800 °C thermal oxidation treatment.

To confirm the established composition of the sample obtained after microwave heating for 60 min and subsequent thermo-oxidative treatment, a comparative analysis of its X-ray diffraction pattern, with the reference X-ray diffraction pattern for these crystalline phases (Figure 2), was carried out. It was found that the main diffraction peaks 2θ 23.9°, 27.0°, 33.8°, 34.3°, and 48.9° corresponded to wollastonite CaSiO_3_ and combeite Na_4_Ca_4_Si_6_O_18_ phases. The remaining peaks have insignificant intensity, so they can be considered as a background.

To visualize the crystal structure of the Na_4_Ca_4_Si_6_O_18_ composite, its structural 3D model was built (Figure 2, inset) using the VESTA software (ver. 3.5.8) [50]. The unit cell parameters coincide with the calculated ones and correspond to the hexagonal form a = b = 10.464 Å, c = 13.176. Combeite belongs to chain silicates, and calcium and sodium have an octahedral configuration. Silicon-oxygen tetrahedrons are joined together by a common oxygen atom at the top of the tetrahedron, while cationic octahedrons are joined to each other and anionic groups via common ribs, or, less often, via vertices. The rule of maximum dense packing is not observed, so there are vacancies in the structure, which can act as sorption centers of the material.

**Figure 3:**
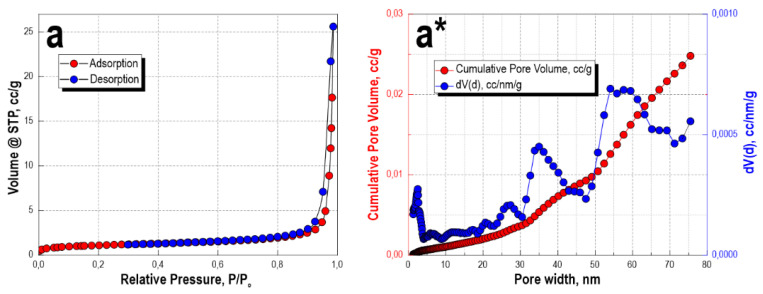
Low-temperature nitrogen sorption-desorption isotherms (**a**) and DFT-calculated pore size distribution histograms (a*) for the sample material obtained after the microwave and thermal oxidation treatments.

The low-temperature sorption-desorption isotherm of nitrogen, for the sample obtained after microwave heating for 60 min and subsequent thermal oxidation treatment, corresponds more to type II, according to the IUPAC classification. This type of isotherm is characteristic of nonporous or macroporous materials of corpuscular structure, wherein the shape and size of pores are heterogeneous (Figure 3a). The isotherm has insignificant sorption hysteresis, which also allows it to be compared with type IV isotherms. This indicates the presence of a small number of micro- and mesopores. This is confirmed by DFT simulation data (Figure 3a*), where it is shown that a pore volume of 0–10 nm corresponds to −0.01 cc/g, a pore volume of 25 nm corresponds to 0.005 cc/g, and 35 nm corresponds to 0.015 cc/g. The main pore volume for this sample lies in the range 50–60 nm—0.02 cc/g; the insignificant presence of micro- and mesopores is present, but without their narrow size distribution. The specific surface area of the sample is 3.7 m^2^/g. Obviously, the bulk of the porous volume belongs to the macropores larger than 100 nm, which are characteristic of the sea urchin skeleton structure and cannot be determined by this method.

**Figure 4:**
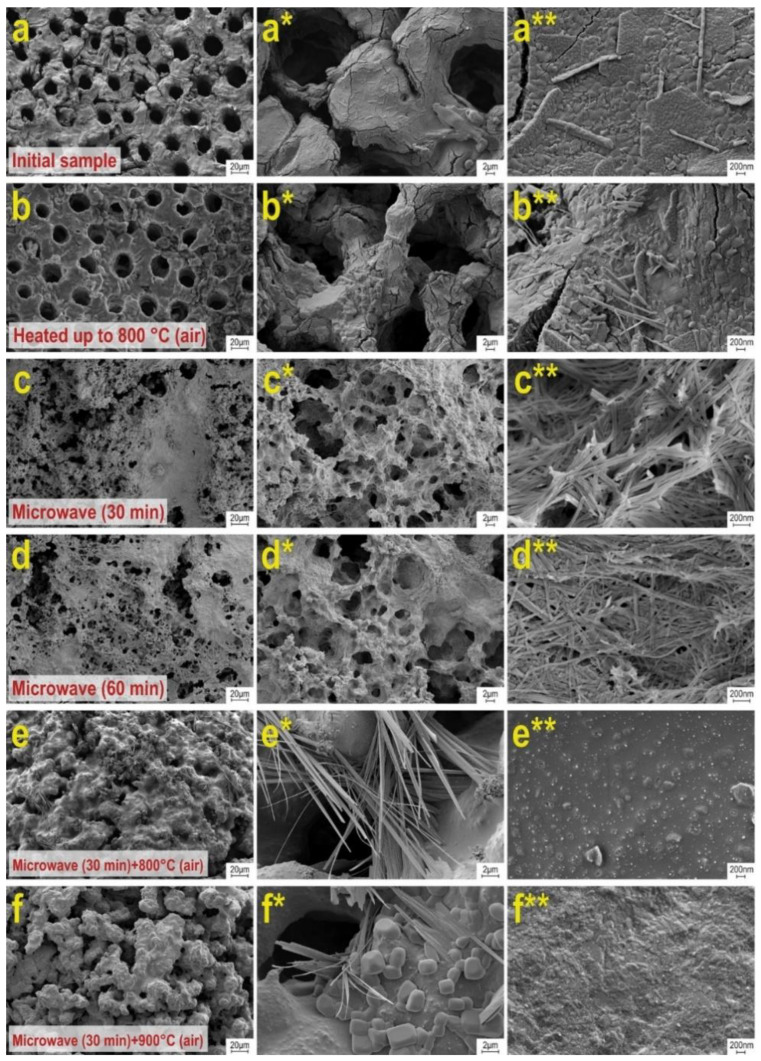
SEM images of the samples: (**a**–a**) original sea urchin skeleton; (**b**–b**) original sea urchin skeleton, calcined at 800 °C in air; (**c**–c**) sample after 30 min microwave synthesis; (**d**–d**) sample after 60 min microwave synthesis; (**e**–d**, **f**–e**) samples obtained by microwave synthesis after additional thermal oxidation treatment at 800 °C.

According to REM data, the surface morphology of the original sea urchin skeleton is represented by a developed macroporous structure (Figure 4a–a**). The surface is rough. Thermo-oxidation treatment of this sample at 800 °C has little effect on the morphology, but some deformation changes in the porous framework, with the appearance of defects in the form of cracks, are observed (Figure 4b–b**). Samples synthesized under microwave heating conditions are characterized by significant destruction of the porous inorganic framework, regardless of the respective treatment times of 30 and 60 min (Figure 4c–c**, d–d**). The porous mass is destroyed, with partial sintering (collapsing) of pores and disordering in size. At the same time, the morphology of the framework changes dramatically, and is represented as a densely packed needle-like structure. This structure corresponds to the structure of wollastonite. Additional heat treatment of these samples at 800 °C is accompanied by sintering of the needle structure and the formation of monolithic agglomerates. However, the macroporous structure and needle formations partially remain (Figure 4e–e** and f–f**).

**Figure 5:**
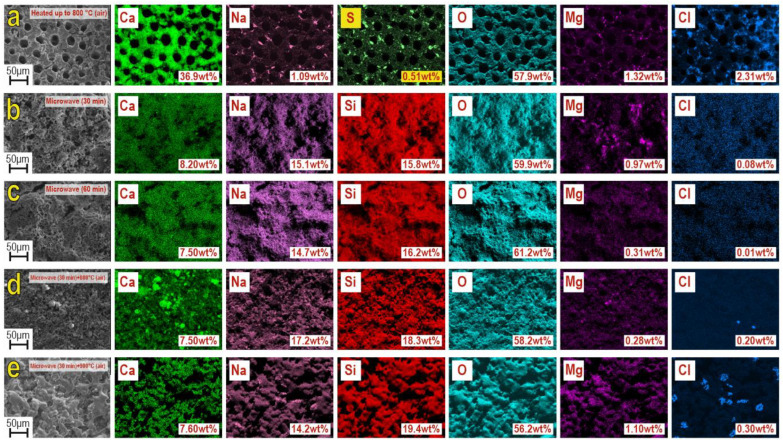
EDS analysis of the samples: (**a**) original sea urchin skeleton, calcined at 800 °C in the air; (**b**) sample after 30 min microwave treatment; (**c**) sample after 60 min microwave treatment; (**d**,**e**) samples obtained by microwave synthesis after additional thermal oxidation treatment at 800 °C.

Qualitative and quantitative EDS analysis (Figure 5) established that the composition of samples included the main elements Ca, O, Si, Na, Mg, evenly distributed on the locally investigated surface. Besides, it contained Mg, Cl, and S in insignificant amounts. The presence of these elements in the sea urchin skeleton is determined by its mineral nature. The exception is Si, which is introduced in the composition synthesis as a part of precursors, according to Equation (1), similarly to Na. These elements represent the basis of the composition of the obtained composite material, wollastonite and combeite phases. The results were confirmed by XRF, shown above (Figure 1 and Figure 2). The quantitative ratio of Ca, Na, Si, and O, in all synthesized samples, corresponds to the newly formed wollastonite and combeite phases.

**Figure 6:**
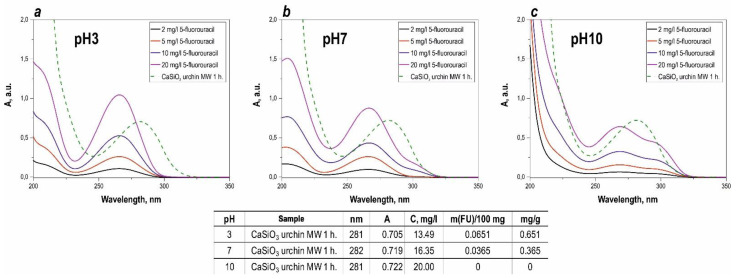
Light absorption spectra of the calibration solutions, and of the solution in the presence of the synthesized calcium silicate composite material at different pH ((**a**)—pH = 3, (**b**)—pH = 7, (**c**)—pH = 10), specially added table to study sorption values of 5-FU in more detail.

The sorption activity of the synthesized calcium silicate composite to adsorb the drug 5-fluorouracil was evaluated at various pH. When the fluorouracil solution was photometrically analyzed, a characteristic absorption peak was observed at 265 nm (Figure 6). It was found that this peak, for the drug solution in contact with the studied composite for 3 days, shifted, relative to the pure substance dissolved in distilled water, which was determined by the influence of the environment created by the composite. There was no difference in the sorption activity when comparing the materials obtained with different treatment times under microwave heating and subsequent thermal oxidation treatment. The highest sorption capacity of the synthesized composite was 12.3 mg/L, with respect to 5-fluorouracil. It is also shown that 5-fluorouracil, at alkaline pH, is not sorbed onto the inorganic sea urchin matrix, as shown in Figure 6c. There is a significant overestimation of the optical density, so it was not possible to measure.

**Figure 7:**
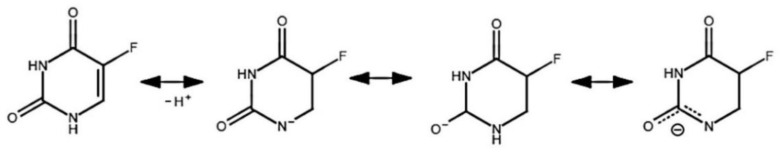
Ionization of the 5-fluorouracil molecule.

The primary mechanism of 5-fluorouracil adsorption to a calcium silicate surface is thought to be hydrogen bonding, due to an unshared electron pair on the oxygen atom of the silicate anion, and mobile hydrogen atoms at N1 and N3 [51]. The possibility of an electrostatic adsorption mechanism is limited by the pH range, at which the adsorbent and adsorbent surfaces have opposite charges. It was shown in [52] that the surface of wollastonite has a significant negative charge, only when the pH increases above 6, which is confirmed by the most complete adsorption of Pb, Cu, Ba, Ca, and Mg metal cations [52,53,54]; however, at high pH equilibrium, deprotonation of 5-fluorouracil shifts towards the negatively charged particles (Figure 7), leading to reduced adsorption, due to repulsive forces of homonymous charges.

In addition, at pH > 9, 5-fluorouracil is hydrolyzed to urea, fluoride, and aldehyde. This hydrolysis is enhanced by increasing the pH and temperature. Some of the urea formed during hydrolysis reacts further to form ammonia and carbon dioxide. Therefore, sorption is not possible at higher pH values.

According to the experimental data presented in the table on Figure 7 the maximum value of the sorption capacity is reached at pH = 3 (0.651 mg/g). At pH = 10, the preparation is not sorbed by the matrix surface. Carrying out the sorption at lower pH values allows a significant increase of the sorption capacity, compared to neutral pH values.

### Discussion Analysis of Drug Release Kinetics

Separation into 2 stages is necessary. As shown from both figures, the release profile of 5-FU from glass nanoparticles had three stages: an initial fast-release stage, a second stage of slower release, and the slowest stage of release. According to the correlation coefficients, the highest Rc value is achieved with the Higuchi model (Table 1).

**Figure 8:**
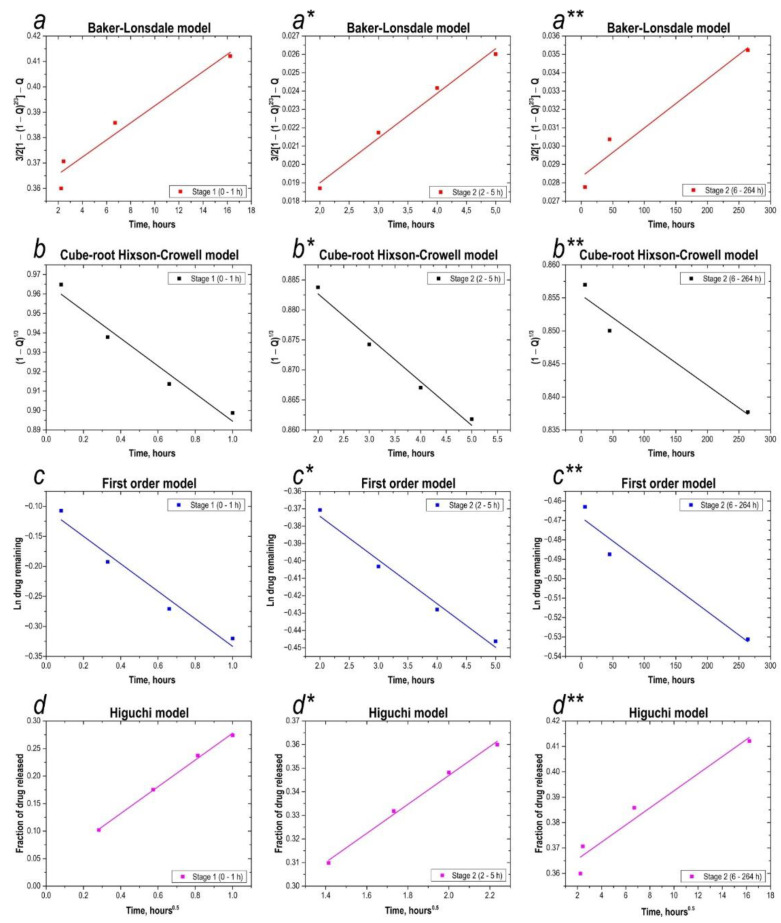
Analysis of drug release kinetics. (**a**–a**)—application of the Baker-Lonsdale model to both stages of the 5-fluorouracil release profile; (**b**–b**)—application of the Hixson-Crowell model to both stages of the 5-fluorouracil release profile; (**c**–c**)—application of the first-order model to both stages of the 5-fluorouracil release profile; (**d**–d**)—application of the Higuchi model to both stages of the 5-fluorouracil release profile.

Modelling the drug release profile using different mathematical models (Figure 8, all) in this work showed that the quality of fit for the first stage was always higher than for the second stage, i.e., higher for the first stage than for the second stage. This observation is consistent with previous studies, which have reported that significant deviation is possible beyond 50% release [43,44,45]. Regression analysis using different models showed that the drug release profile from a loaded inorganic 5-fluorouracil sea urchin in the first and second stages was best described by the Baker-Lonsdale and Higuchi models, as the approximation of the release profile curve showed higher linearity than other models. However, the other models used in this work were also applicable, as they showed fairly good linearity with the release profile, with relatively high correlation coefficients, especially in the first stage. (Figure 8 d–d**).

A significant part of the drugs were released in the first 6 h, when the release of 37% of 5-fluorouracil was observed. In the next 258 h, the sustained release of drugs continued at a much slower rate, with an additional 5.2% of 5-fluorouracil. Matrix degradation was estimated by the content of calcium ions on days 1, 3, and 7 in SBF solution, imitating the ionic composition of the inorganic component of human blood plasma. According to the data of the experiment, the highest concentration of calcium was fixed after 1 day of being kept in the solution. After that, the amount of Ca^2+^ ions leached with the time (34.1, 27.2, and 25.3 mg/L, respectively), which can be explained by less intensive leaching of ions from the surface of the particles. Agglomeration of the particles was observed when the powder ceramics were in the solution, which also slowed down the rate of bioresorption.

To describe calcium leaching, a model of change in the chemical stability of inorganic matrices was used, based on the calculation of the leaching rate using the formula:

$$R_{n}^{i}=\frac{a_{n}^{i}}{A_{0}^{i}St_{n}}$$

were $a_{n}^{i}$—the mass, g, of a single ion (or mixture of them) leached in a given time interval;

$A_{0}^{i}$—the mass concentration, g/g, of the ion (or mixture of them) in the original sample;

S—the area of the open geometric surface of the sample, cm^2^;

$t_{n}$—the duration of the n-th leaching period, days.

**Figure 9:**
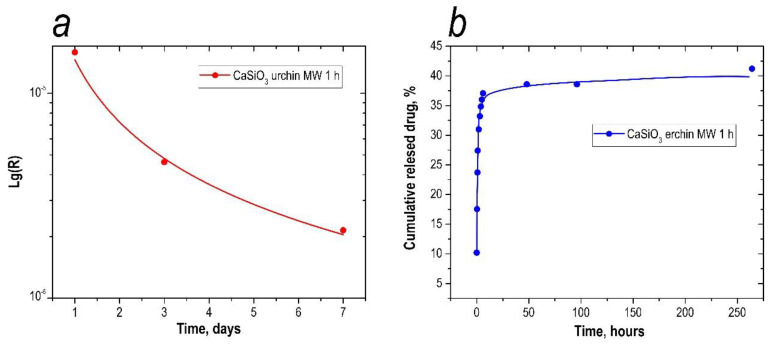
Calcium degradation from the inorganic matrix (**a**) and percentage of 5-fluorouracil leached from the inorganic matrix (**b**).

The concentration of ions in the solution was determined by atomic adsorption. The descending shape of the calcium leaching rate plot also confirms the resorption of the matrix over time (Figure 9a).

Drug release studies were performed in a mildly acidic environment (pH = 5), simulating the pH of the affected tumor area.

The total amount of drug loaded into the sample was determined by cumulative adsorption. For matrix loading, the sorption method was used at pH = 3, as the sorption capacity is maximal under these conditions (0.650 mg of 5-fluorouracil per 1 g of solid). This amount was taken as 100% in the release study, and was used to calculate the percentage of amount released at each time interval. From the UV spectroscopy results, time-dependency curves of the amount of 5-fluorouracil released were obtained. The results are presented in Figure 9b.

As can be seen in Figure 9b, at pH = 5, a large proportion of the drug was released during the first hour, with 27% of 5-fluorouracil being released (Figure 9b). During the next 3 h, drug release continued at a slower rate, with an additional 9% desorbed. The analytical signal of 5-fluorouracil was fixed for up to 11 days. We assumed that the drug release continued for a longer period of time, but the recording of the 5-fluorouracil content in the sample was limited by the instrumentation available in the laboratory. Thus, the total percentage of 5-fluorouracil leached from the inorganic matrix was 41%, or 0.267 mg/g.

## 4. Conclusions

An original synthesis of a composite based on crystal wollastonite CaSiO_3_ and combeite Na_4_Ca_4_(Si_6_O_18_) using a sea urchin skeleton *Mesocentrotus nudus* by microwave heating in hydrothermal conditions was carried out. It was shown that the sea urchin skeleton acted as a raw material component in the form of CaCO_3_ (source Ca^2+^), to initiate the reaction of calcium silicate formation during the synthesis of the material. XRF data showed that the microwave heating times of 30 and 60 min did not affect the phase composition of the obtained composite. At the same time, thermal oxidation post-treatment of the material led to an increase in the degree of crystallization of the main phases of wollastonite CaSiO_3_ and combeite Na_4_Ca_4_(Si_6_O_18_). REM data showed that microwave heating under hydrothermal conditions promoted the formation of a needle-like structure of CaSiO_3_ wollastonite. The sea urchin skeleton played the role of a matrix for the formation of a porous inorganic framework. At the same time, thermo-oxidative post-treatment led to the destruction of the porosity and sintering of the needle structure of the composite into a monolithic form. According to the data of EDS analysis, the chemical composition of the composite was uniform in volume, with a slight admix of Mg, Na, and Cl as elements of the composition of the initial sea urchin skeleton. The sorption capacity of the composite, with respect to 5-fluorouracil, was 12.3 mg/L, regardless of the time of its microwave synthesis and thermal post-treatment in the air. The study results show that the obtained composite is a promising carrier for the targeted delivery of chemotherapeutic drugs. It was decided to conduct bio-tests of the composite in the in vitro and in vivo models in further research. Drug-loaded nanoparticles, which can release therapeutic doses of 5-FU over a longer period of time than other systems described in the literature, can prevent tumor recurrence after resection.

## Figures and Tables

**Table 1:** Analysis of drug release kinetics with correlation coefficients. Application of Higuchi model, first-order model, Beker-Lonsdale model, and Hixson-Crowell model.

Mathematical Models	Stage 1 (0–1 h)		Stage 2 (2–5 h)		Stage 3 (6–264 h)	
	Correlation Coefficient (𝑅_𝐶_)	Release Rate Constant	Correlation Coefficient (𝑅_𝐶_)	Release Rate Constant	Correlation Coefficient (𝑅_𝐶_)	Release Rate Constant
Higuchi	0.9975	k_1_ = 0.24 h^–0.5^	0.9951	k_1_ = 0.06 h^–0.5^	0.9958	k_1_ = 0.30 × 10^−2^ h^–0.5^
First-order	0.9694	k_2_ = 0.23 h^–1^	0.9842	k_2_ = 0.03 h^–1^	0.9518	k_2_ = 0.02 × 10^−2^ h^–1^
Baker-Lonsdale	0.9951	k_3_ = 0.01 h^–1^	0.9883	k_3_ = 0.24 × 10^−2^ h^–1^	0.9562	k_3_ = 0.30 × 10^−4^ h^–1^
Hixson-Crowell	0.9659	k_4_ = 0.07 h^–1^	0.9833	k_4_ = 0.73 × 10^−2^ h^–1^	0.9505	k_4_ = 0.70 × 10^−4^ h^–1^

## Data Availability

Not applicable.
